# Genealyzer: web application for the analysis and comparison of gene expression data

**DOI:** 10.1186/s12859-023-05266-4

**Published:** 2023-04-17

**Authors:** Kristina Lietz, Babak Saremi, Lena Wiese

**Affiliations:** 1 Research Group Bioinformatics, Fraunhofer ITEM, Hannover, Germany; 2 Institute of Computer Science, Department of Mathematics and Computer Science, Goethe University, Frankfurt, Germany

**Keywords:** Microarray, Webapplication, Gene expression

## Abstract

**Background:**

Gene expression profiling is a widely adopted method in areas like drug development or functional gene analysis. Microarray data of gene expression experiments is still commonly used and widely available for retrospective analyses. However, due to to changes of the underlying technologies data sets from different technologies are often difficult to compare and thus a multitude of already available data becomes difficult to use. We present a web application that abstracts away mathematical and programmatical details in order to enable a convenient and customizable analysis of microarray data for large-scale reproducibility studies. In addition, the web application provides a feature that allows easy access to large microarray repositories.

**Results:**

Our web application consists of three basic steps which are necessary for a differential gene expression analysis as well as Gene Ontology (GO) enrichment analysis and the comparison of multiple analysis results. Genealyzer can handle Affymetrix data as well as one-channel and two-channel Agilent data. All steps are visualized with meaningful plots. The application offers flexible analysis while being intuitively operable.

**Conclusions:**

Our web application provides a unified platform for analysing microarray data, while allowing users to compare the results of different technologies and organisms. Beyond reproducibility, this also offers many possibilities for gaining further insights from existing study data, especially since data from different technologies or organisms can also be compared. The web application can be accessed via this URL: https://genealyzer.item.fraunhofer.de/. Login credentials can be found at the end.

## Introduction

Gene expression analysis is used to investigate biological processes in a wide variety of circumstances, such as the identification of molecules in metabolism, in tumor research, or the search for target genes in drug development. The rise of different technologies such RNA-seq and popular platforms like Agilent and Affymetrix gave scientist a variety of different methods to study differential gene expressions. [1–3]. However, analyzing the increasing data volumes has become challenging in recent years. Studies as shown by Vescovo et al. demonstrate that different platforms and techniques can yield differences in results [4]. In addition, the cost reduction of sequencing was reduced over the years which in turn increased the amount of data produced. Analyzing the high abundance of data often requires expert knowledge and programming skills, which in return generates the need for bioinformatics analysis tools that can alleviate some of the challenges presented [5]. The scientific community implemented guidelines to orient their analysis on e.g. the MaEndToEnd workflow [6]. Although these guidelines help to address best practices, they do not fully alleviate the programming challenges since adaptation of the published code to the individual data is still required. Since available data is generated with different technologies and processed by different software (e.g. limma [7]), the comparison in order to integrate the results of several Differential Gene Expression (DGE) analyses is hence not straightforward [8].

Besides the differences in technologies, different platforms also use their own identifiers for genes and thus increasing the difficulty to compare results. By using old results and comparing them to newer ones re-doing an experiment can be avoided. Furthermore, some studies use surrogate models to investigate an organism that has not be sufficiently annotated. Comparing results of closely related organisms, can help to generate new insights. In addition, using surrogate models and already published data could also be used for comparing DEGs between e.g. human and mice to investigate the usefulness of a possible animal experiment beforehand.

This paper presents a web application that provides a user-oriented web service for the basic steps of DGE analyses, comparison of multiple DGE analyses, and GO based enrichment analyses.

## Background

The popular use of DGE analysis sparked the creation of many different tools. However, many of the tools present additional hurdles for scientist that are missing programming backgrounds.

Tools like NetworkAnalyst and miRNet 2.0 [9] provide a web application for microRNA network analysis that gives users access to their platform without the need for installing and maintaining the software. However, in order to use miRNet 2.0 users are required to upload their data in a specific file format with a defined structure that is different from data provided by gene expression data platforms like the Gene Expression Omnibus (GEO) [10, 11]. This forces users to convert their data programmatically.

Other tools are commonly not provided as a web application which in return has to be installed by the user. Installing bioinformatics tools often require either a specific platform e.g. servers or a specific operation system (Linux). Examples are: Chipster [12] provides over 500 analysis tools for different types of gene expression data but requires complex installation on servers, if no user account is purchased. ArrayTrack [13] offers a toolbox and has the additional feature to host and manage gene expression data, but requires a Windows operating system with a Java environment. GEPAT [14] also provides gene expression analysis with the added benefits of biological interpretation. However, it is recommended by GEPAT to carry out the installation of the necessary libraries and the tools by a system administrator.^1^

Lastly, tools like Gecko [15], MMIA [16] and GEPAS [17] have interesting approaches, like sharing results with users (Gecko), combining analysis of miRNA and mRNA (MMIA) or clustering approaches (GEPAS). However, these tools were not updated for a considerable time or are not available anymore and could thus lead to problems of reproducibility and robustness.

For some users it might be more intuitive to use to official tools provided by the platforms that either generate their data or host them. The Transcriptome Analysis Console (TAC) software [18] is provided by Affymetrix Inc., the manufacturing company of the Affymetrix microarray chips. A variety of analyses are possible with this, but the installation on the client is mandatory. Another disadvantage is that the analysis of Agilent data is not possible at all. GEO2R [10] offers an option for gene expression analysis available on the website of the GEO database. Local files cannot be uploaded and only one biological group per sample can be specified, which limits the possible analyses. In addition, the analysis of two-channel Agilent data is not possible.

The tools presented thus far, mostly focus on analyzing data of a single gene expression experiment. The comparison of multiple analyses is only possible to a limited extent. However, the tools that are most comparable to the goals of our approach are TAC, GEO2R and NetworkAnalyst. In the chapter chapter:Requirements, we compare the features between our and these three applications again in more detail.

This article aims to address these limitations by presenting a web application that provides an end-user oriented web interface for the basic steps of DGE analyses, consisting of data upload, preprocessing which includes quality control and the specification and execution of actual DGE analyses. Additionally, the comparison of multiple DGE analyses and GO based enrichment analyses can be realized in the application.

Our main contributions in this paper are the following:

- We present the technical features of our DGE analysis oriented to the MaEndToEnd workflow as a web application
- We provide an in-depth discussion of two use cases (Inflammatory Bowel Disease and Rhino Virus)
- We provide a comparison of our tool to existing tools for gene expression analysis

## Methods

In this section we discuss the main technical components of our Genealyzer Web application. We present a range of available features including the following: (1) Uploading data is enabled from the local storage and directly from the GEO repository [10, 11]; (2) Depending on the microarray chip used, a preprocessing algorithm can be selected; (3) A Sample and Data Relationship Format (SDRF) file [19] for defining the experimental factors can either be uploaded or generated inside the application; (4) The application allows a variety of customized visualizations for quality control and analysis results; (5) Furthermore, after quality control the filtering of outlier samples is possible. Further details on the implementation from a software engineering perspective are reported in [20].

### User interface and architecture

The application was implemented using the programming language R (version 4.1.2). R provides an environment for the analysis and graphical visualization of data. R was chosen for the implementation because the open-source software project Bioconductor [21] provides many R packages for the analysis of gene expression data. Although these could be used in other programming languages, the application’s complexity can be reduced by limiting it to one programming language. In order to implement the web application, the R package shiny (version 1.7.1) [22] was used, which provides a framework for building interactive web apps.

**Fig. 1:**
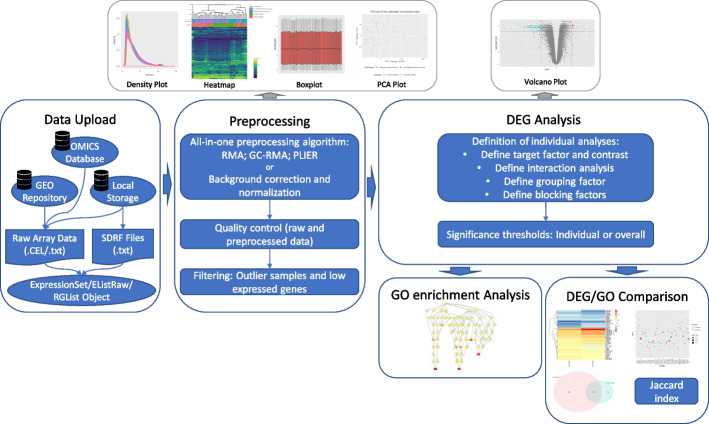
Pipeline of the web application

User interaction without programming skills is enabled by an appropriate interface design. To increase usability and learnability, one tab was created for each main step of the pipeline visualized in Fig. 1. The tabs have a consistent structure: A sidebar allows defining the main input parameters of the respective step, such as selecting a preprocessing algorithm or the definition of analyses. In the main panel, results, plots, and options for detailed analyses are displayed. For steps involving choices, default values, which are often found in literature, are specified. An example of this is the selection of the RMA algorithm for the preprocessing of Affymetrix data.

Another non-functional requirement was the reproducibility of the analysis steps. For fulfilling this, a logging file can be downloaded. This file tracks the essential analysis steps, especially the parameters defined by the user.

The main steps of the application pipeline are visualized in Fig. 1. Many steps of the pipeline are derived from the MaEndToEnd Workflow presented by Klaus and Reisenauer [6]. First, the user can upload the raw data and an SDRF file for each source. Alternatively, the latter file can also be defined via the user interface (UI) within the application. In the preprocessing step, the user can choose an all-in-one preprocessing algorithm for Affymetrix data or a background correction and a normalization algorithm for Agilent data. Moreover, the quality control of the raw and the preprocessed data can be performed. Detected outlier samples, as well as lowly expressed genes, can be filtered. Next, the user can define an individual number of flexible DGE analyses and set the significance thresholds. The performed DGE analyses of data produced with the same microarray chip can be compared via different visualizations and by calculating the Jaccard index on the sets of expressed genes. In addition, GO-based enrichment analyses can be performed. For all steps, the generated plots and the result tables can be downloaded.

### Data upload

Our web application allows the upload of the raw files both, from the local storage and directly from the GEO platform using the GEOquery package (version 2.62.2) [23]. To use the files from the GEO repository, the GEO series accession number (GSE) must be entered, which identifies the series. It is possible to filter the samples from the defined series since not all samples may be of interest. The user can define as many sources as desired. After uploading, the contents of the SDRF files and the metadata of the sources can be verified and modified if necessary. Furthermore, the SDRF file can be downloaded.

Our tool supports the analysis of Affymetrix as well as one- and two-channel Agilent microarray data.

### Preprocessing

Depending on the microarray technology, the data must be loaded differently. First, we consider Affymetrix data. The raw data is in CEL file format, of which three different versions exist. These differ in the structure of the contents and the data format. The format depends on the type of microarray chip used in the experiment [24]. The data of each different microarray chip is stored in a subclass of the ExpressionSet class defined in the Biobase package (version 2.54.0) [25]. For reading in the data, the affy package (version 1.72.0) [26] is preferred over the oligo package (version 1.58.0) [27]. Yet, because new versions of CEL files, like Gene ST arrays, cannot be processed with the affy package, the oligo package is used for reading the data of those chips.

Next, we present the data upload of Agilent data, which is in TXT file format. Here, single-channel experiment designs and two-color experiments have to be distinguished. The data is read in using the limma package (version 3.50.1) [28] in both cases. If the considered experiment is in single-channel design, the data is stored as an object of the EListRaw class. Otherwise, it is stored as RGList.

After the data upload, the user can select between different preprocessing algorithms, plots for the quality control and filter options. First, the uploaded raw data can be filtered by the microarray chip. One source may contain files of different chips, but not necessarily all chips should be analyzed.

We will again first discuss the procedure for Affymetrix data. Depending on the package used for reading the data as an ExpressionSet object, different all-in-one algorithms for the steps background correction, normalization, and summarization are available. If the affy package was used, the options are the RMA algorithm [29], the GC-RMA algorithm [30], and the PLIER algorithm [31]. Otherwise, only the RMA algorithm can be selected since the other ones are not provided by the oligo package.

There are no all-in-one algorithms for preprocessing Agilent data. Hence, the user can select one algorithm for background correction and one for normalization. For the background correction, either no algorithm or the algorithms half, minimum, edwards and normal/exponential distribution can be selected for both kind of experiments. One-channel experiment designs can be normalized using the scale, quantile or cyclic loess algorithm. The algorithms median, loess, print-tip loess and robust spline can be selected for normalize two-color experiments. More information about the different preprocessing algorithms can be found in the limma user guide [32].

The user can visualize the data in various ways for quality control. For each chip type, boxplots and density plots can be generated for the raw data and for the preprocessed data. Moreover, the user can generate clustered heatmaps of the preprocessed data. For this, the user can select which experimental factors should be included in the annotation of the columns. This allows the detection of differences in signal intensity between experimental groups. Furthermore, the user has the possibility to create Principal Component Analysis (PCA) plots of the preprocessed data. The user can choose up to two factors whose groups of samples are visualized in the plot. Of the selected factors, the factor values for which the corresponding samples are shown can also be filtered. This allows the visualization of batch effects.

Based on the quality control plots presented, outlier samples can be removed for further analysis. Additionally, lowly expressed genes can be filtered. For this purpose, a threshold can be set for each chip individually using a histogram of the median intensities. Genes with a lower intensity are filtered out.

We used the ggiraph package (version 0.8.3) [33] for implementing the boxplots and the PCA plots. This package allows the creation of interactive ggplot2 (version 3.3.6) [34] plots. The plotly package (version 4.10.0) [35] provides a similar functionality and was used for the density plots. We implemented the heatmaps using the heatmaply package (version 1.4.0) [36], which allows for interactive heatmaps.

### DGE analysis

The web application allows for the definition of any number of DGE analyses. For this purpose, the user can first define the name of the analysis to simplify the analysis’ recognition. A contrast of an experimental factor between which the DEGs should be identified is defined. If an interaction analysis should be performed, the user selects a second factor and defines the contrast for this as well. In addition, the user can optionally select the value of a third factor, according to which the samples will be grouped. Moreover, blocking factors may be selected. Blocking factors are variables that are expected to have an impact on the result but are not of interest. Thus, differences in expression between the corresponding factor values will be intercepted [6].

A histogram of the distribution of the p-values is plotted for each analysis, with frequencies expected to be very high, near zero and low toward one. The user can verify this step. If the histogram does not meet the expectations, the workflow ends at this point. Possible faults could be incorrect data, incorrect preprocessing, or improperly defined analyses.

If the histogram meets the expectations, the next step is to set the significance thresholds. These define ranges of statistical values calculated during the analysis that identify a gene as differentially expressed. The user can choose whether the thresholds should be defined for all analyses or individually for each analysis. Either the *p*-value or the false discovery rate [37] can be selected as the indicator for the strength of the evidence against the null hypothesis. The null hypothesis in a DGE analysis is that there is no differential expression. Also, the user can define a cutoff value for the absolute log2 fold change, which is applied for both positive and negative fold changes.

Based on the specified values, a volcano plot is generated where user-defined input data of up- and down-regulated genes are colored. The user verifies the results and can adjust the threshold values again if necessary. A table of DEGs with various statistical values is also shown, which can be downloaded if required.

For the implementation of the linear models and the DGE analyses, the limma package (version 3.50.1) provided by Bioconductor was used.

### GO enrichment analysis

Next, the web application allows GO-based enrichment analyses. One DGE analysis can be assessed according to the three GO top ontologies (biological processes, cellular components, and molecular functions [38–40]), or by different algorithms (e.g. classis, elim, weight01, see [41]). The fisher test statistic is used as test statistic for the analysis.

Once all GO analyses are defined, the density of the median intensities of the DEG (foreground genes), all genes and calculated background genes are plotted. The latter is calculated automatically and should show a similar expression behavior as the foreground genes. The user can choose from a multidensity plot, whether a calculated set of genes with a similar behaviour to the DEGs or whether all genes available through the chip should be used as background for the analysis. A table containing the significant GO categories can be downloaded if required. Additionally, the significant categories can optionally be visualized in the context of the GO hierarchy. This graph is created as a PDF file and downloaded automatically.

We used the R package geneplotter (version 1.72.0) [42] for generating the multidensity plot and the topGO package (version 2.46.0) [43] for performing the actual GO analysis.

### DEG/GO comparison

The last main step of the pipeline is the comparison of the resulting DEG’s and GO Terms. This step can also be done independently of the previous steps. The user can perform as many comparisons as necessary. DGE analyses can only be compared with each other, the same applies to GO analyses. When defining a new comparison, a name can be specified first. Then the user determines whether DGE or GO analyses are to be compared. Analyses carried out during the current session and analysis results uploaded as CSV files can be compared with each other. Theoretically, an unlimited number of analyses can be compared, but it should be noted that the plots become confusing if there is too much content. In the next step, the user checks whether the correct columns for ID, name, *p*-value and fold change are selected for all analyses. For GO analyses, the fold change column is not required.

After all desired comparisons have been defined, the user can select individually for each comparison which plots or ratios are to be calculated:

- For DGE analyses
  - Heatmap and scatterplot of log 2 fold change for genes with smallest adjusted *p*-value
  - Venn diagram of up-regulated, down-regulated and all DEG (up- and down-regulated)
  - Table of up-regulated, down-regulated and all DEG in the intersection
  - Jaccard-Index
- For GO analyses
  - Scatterplot of *p*-value for categories with smallest adjusted *p*-value
  - Venn diagram of categories
  - Table of categories in intersection
  - Jaccard index

In Venn diagrams, a maximum of five analyses can be visualised. If more than five analyses are specified, the user can select up to five analyses and update the diagram as often as desired. The Jaccard index can only be calculated for two analyses at a time. We used the packages VennDiagram (version 1.7.3) [44], ggplot2 (version 3.3.6) [34], ggiraph (version 0.8.3) [33] and pheatmap (version 1.0.12) [45] for the implementation of the graphics.

## Results

This chapter presents two case studies that demonstrate the potential of our web application to facilitate comparative analysis of gene expression datasets. The first case study compares the differences in genome-wide expression between two common forms of inflammatory bowel disease–Ulcerative colitis (UC) and Crohn’s disease (CD). The second case study compares human and mouse gene expression data of samples challenged with the rhinovirus.

### Case study I—inflammatory bowel disease

As a case study we present here a genome-wide pathway analysis using gene expression data of colonic mucosa in patients with inflammatory bowel disease. The data of this study is also used in the presentation of the MaEndToEnd Workflow [6], which offers a guideline for a step-by-step DGE analysis using Bioconductor R packages. The study aims to explore the differences in genome-wide expression between UC and CD.

**Fig. 2:**
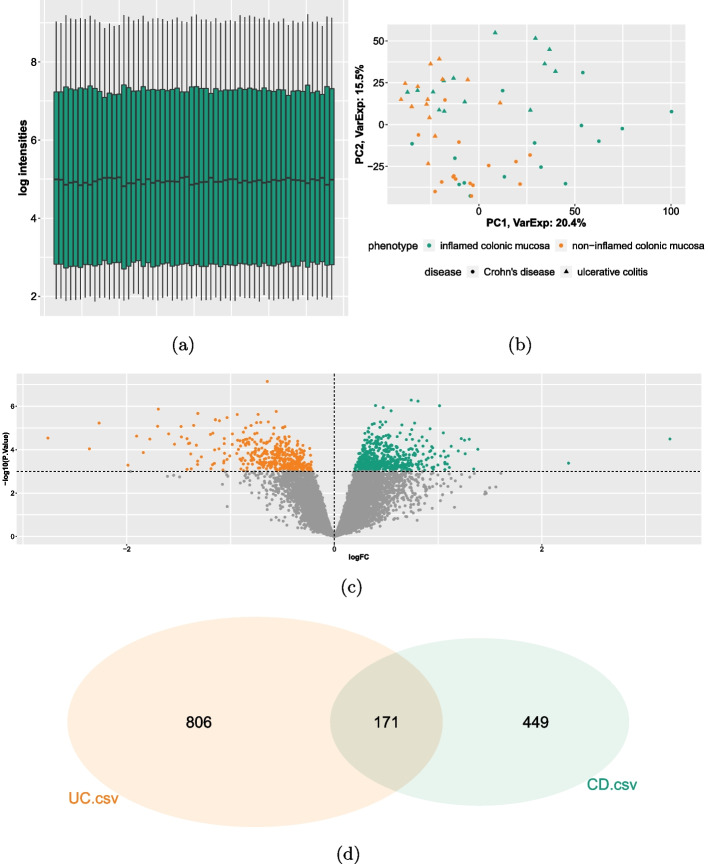
Results of case study I. **a** Boxplot of the preprocessed data for quality control shows that the log intensities are overall evenly distributed. **b** PCA plot helps to identify outlier samples and visualizes different biological groups based on the factor values via color and symbol codes. **c** Volcano plot shows the result of the UC DGE analysis, where up- and down-regulated genes are highlighted. **d** Venn diagram of all DEG shows the intersection between the DGE analyses to compare

We downloaded the data and the SDRF file from the ArrayExpress platform and then uploaded it in the web application from our local storage. Then, we preprocessed the data using the all-in-one algorithm RMA and we verify the quality of the raw and the prepocessed data using the plots described in chapter Preprocessing. As an example, the boxplot and PCA plot of the preprocessed data is shown in Fig. 2a and 2b. Based on these, no outlier samples are detected. For filtering lowly expressed genes we set a minimum threshold of a median intensity of 4, similar to [6].

We define two DGE analyses, where the expression between inflamed and non-inflamed tissues is to be compared. Because we aim to investigate the differences between UC and CD, we group the samples according to these diseases. As blocking factor, we set the factor ‘individual’, meaning that differences in the expression that are based on different human donors should be excluded. Klaus and Reisenauer chose a *p*-value cutoff of 0.001 for both analyses, so we define the same threshold to keep comparability. We find 977 DEG for UC and 620 DEG for CD. The volcano plot visualizing the DEG of the CD analysis is shown in Fig. 2c. Hence, we identified slightly more DEG than presented by Klaus and Reisenauer, who found 944 DEG for UC and 577 DEG for CD. The differences may be based on differences on rounding. Comparing both analyses, we find 68 up-regulated, 103 down-regulated and accordingly 171 DEG in total in the intersection of both. Figure 2d shows the Venn diagramm of all DEG for visualization of the comparisons. This leads to a Jaccard-Index of 0.0882 for the set of up-regulated, 0.1573 for the set of down-regulated and 0.1199 for all DEGs.

Next, we conduct GO enrichment analyses for both DGE analyses. We chose ‘Biological process’ as top ontology and the ‘elim’ algorithm. In both analyses we choose the calculated set of genes as background. When comparing both GO analyses in the next step, we find 19 GO categories in both results, meaning that these categories are in the top 100 significant categories for both DGE analyses.

### Case study II—rhinovirus

In our second case study, we want to illustrate the advantages of the comparison feature. We compare human data (GSE137905) from the Affymetrix chip HG-U13_Plus_2 with mouse data (GSE126832) produced with the Affymetrix chip MTA-1_0. After quality control, we first performed the DGE analyses, comparing samples challenged with rhinovirus for 24 h with control samples for the human data set. For the mouse data, we compared infection and control samples with media control samples. Thus, for both organisms, we analyzed the DEG between healthy Precision Cut Lung Slices (PCLS) and PCLS infected with rhinovirus. With these results, we performed GO analyses for the biological function and compared the results in the comparison tab. One interesting finding is the the apperance of NF-kappaB (nuclear factor k-light-chain-enhancer of activated B cells) and the tumor necrosis factor as up regulated genes. Both were shown by Laza-Stanca et. al [46] to be linked with the rhinovirus.

**Table 1:** Intersection of significant regulated GO categories in Human as well as in Mouse PCLS after Rhinovirus infection (case study II)

GO.ID	Term
GO:0001916	Positive regulation of T cell mediated cytotoxicity
GO:0001961	Positive regulation of cytokine-mediated signaling pathway
GO:0002230	Positive regulation of defense response to virus by host
GO:0002250	Adaptive immune response
GO:0002474	Antigen processing and presentation of peptide antigen via MHC class I
GO:0030593	Neutrophil chemotaxis
GO:0032728	Positive regulation of interferon-beta production
GO:0032729	Positive regulation of type II interferon production
GO:0032731	Positive regulation of interleukin-1 beta production
GO:0032760	Positive regulation of tumor necrosis factor production
GO:0034341	Response to interferon-gamma
GO:0035458	Cellular response to interferon-beta
GO:0043123	Positive regulation of I-kappaB kinase/NF-kappaB signaling
GO:0043330	Response to exogenous dsRNA
GO:0045071	Negative regulation of viral genome replication
GO:0045087	Innate immune response
GO:0045089	Positive regulation of innate immune response
GO:0045824	Negative regulation of innate immune response
GO:0050729	Positive regulation of inflammatory response
GO:0050830	Defense response to Gram-positive bacterium
GO:0051092	Positive regulation of NF-kappaB transcription factor activity
GO:0051607	Defense response to virus
GO:0060333	Type II interferon-mediated signaling pathway
GO:0060339	Negative regulation of type I interferon-mediated signaling pathway
GO:0071222	Cellular response to lipopolysaccharide
GO:0071346	Cellular response to interferon-gamma
GO:0071347	Cellular response to interleukin-1

**Fig. 3:**
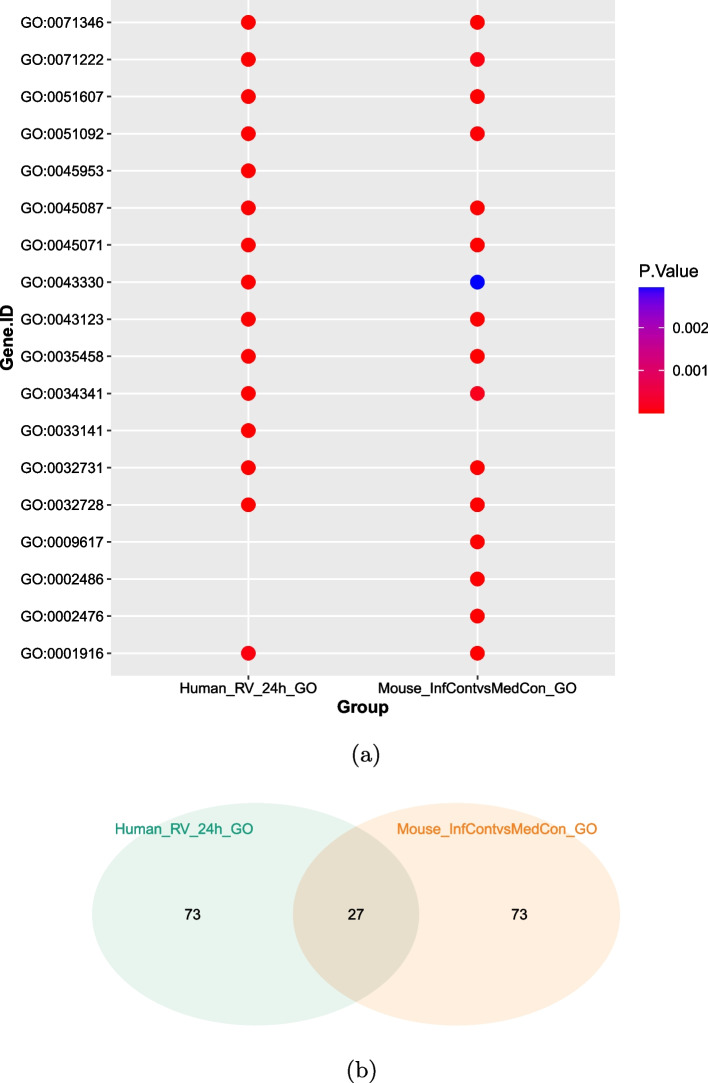
Results of case study II. **a** Scatterplot of the most significant GO categories based on the *p*-values shows the differences between the individual studies. **b** Venn diagramm shows the intersection of the significant GO categories

Figure 3 visualizises the comparison results. First, the Venn diagram shows that around 25% of the significant categories are significant for both analyses. The scatterplot shows the 10 most significant GO categories of both, the human and mouse data. Missing dots mean, that the respective category is not in the most 100 significant categories of the analysis, so for example the category GO:0045953 (negative regulation of natural killer cell mediated cytotoxicity) is only significant for human, but not for mouse. The colors indicates how significant the categories are based on the *p*-value, but regarding to the color scale all shown categories are very significant, since the *p*-value is 0.003 or lower. In general, the scatterplot shows that the majority of the top ten significant categories is significant for both analyses.

Table 1 includes the GO categories which are identified as significantly regulated for human and mouse. Having a close look on these categories, there are some interesting results which enable assessments about for example the usage of animal experiments with mice for the development of drugs against the rhino virus. Also a deeper understanding of the biological processes after an infection can be achieved.

## Discussion

The increasing amount of sequencing data that is available today poses a problem for scientist that do not have a programming background. One popular way to handle the increasing amount of gene expression data is the employment of UIs which help scientists to alleviate the programming challenges for statistical and exploratory analysis. Even though an UI can reduce the programming complexity, it is worth mentioning that the reduction of complexity comes at the price of flexibility. By using an UI all the necessary steps have to be included were as by programming pipelines by hand, changes can be done more easily.

### Requirement analysis

Based on user interviews, we obtained several requirements for our tool. We briefly describe these requirements in more detail. The application should enable to analyze Affymetrix as well as one-channel and two-channel Agilent data. The upload of files in the original format (CEL or in original TXT format) and the upload of SDRF files from the local storage should be possible. For Affymetrix data, it should be possible to apply an all-in-one preprocessing algorithm on the raw data. For quality control, the generation of boxplots of the raw and preprocessed data, as well as PCA plots and clustered heatmaps of the preprocessed data should be offered by the application. Based on this, outlier samples should be filterable. The user should be able to define a dynamic number of flexible DGE analyses as described before. It should be possible to define individual significance thresholds for each defined analysis. The DGE analysis result should contain at least a volcano plot and a table of the defined DEG including the *p*-value, adjusted *p*-value and the log or linear fold change. It should be possible to compare DGE analyses. In particular, the ID on which the analyses are to be compared should be selectable. Therefore, the result table of the DGE analysis of our application already includes the Ensembl stable ID, which enables the comparison of analyses between different microarrays, even of different organisms. Moreover, the application should offer GO based enrichment analyses and the comparison of these. Non-functional requirements are the implementation as a web application to prevent the installation of the software and the reproducibility of the defined analysis by storing the defined parameters.

As mentioned in the section allowing early conclusions abourefsec:background, there is a number of open-source tools for analyzing microarray data available. Although most tools have their own benefits and weaknesses, we identified three tools to be most in line with our requirements of usability and the basic DGE analysis functionalities described by our user interviews. In Table 2 we show the comparison of functionalities between the TAC software [18], GEO2R [10], NetworkAnalyst [47] and our tool. A check ($\checkmark$) means, that the requirement is either completely fulfilled or that the functionality may be added by some additional approach. A cross ($\times$) means, that the requirement is only partly or not at all fulfilled. The comparison of multiple DGE analyses and thus the comparison [48, 49] of gene expression between, for example, different diseases or organisms is not sufficiently enabled with existing tools. Moreover, to the best of our knowledge, enrichment analyses, such as the classification of DEGs according to the Gene ontology (GO), are often not included in most tools, or only in a complicated way.

**Table 2:** Evaluation of meeting the requirements graded ûhighö in comparison to existing tools for gene expression analysis

Requirement	TAC	GEO2R	NetworkAnalyst	Our tool
Analyze affymetrix data	$\checkmark$	$\checkmark$	$\checkmark$	$\checkmark$
Analyze one-channel agilent data	$\times$	$\checkmark$	$\checkmark$	$\checkmark$
Analyze two-channel agilent data	$\times$	$\times$	$\checkmark$	$\checkmark$
Upload files in original format	$\checkmark$	$\checkmark$	$\times$	$\checkmark$
Upload SDRF files	$\checkmark$	$\checkmark$	$\checkmark$	$\checkmark$
Upload from local storage	$\checkmark$	$\times$	$\checkmark$	$\checkmark$
All-in-one preprocessing (Affymetrix)	$\checkmark$	$\checkmark$	$\times$	$\checkmark$
Boxplots	$\checkmark$	$\checkmark$	$\checkmark$	$\checkmark$
PCA plots	$\checkmark$	$\times$	$\checkmark$	$\checkmark$
Clustered heatmaps	$\checkmark$	$\times$	$\checkmark$	$\checkmark$
Filter outlier samples	$\checkmark$	$\times$	$\times$	$\checkmark$
Flexible DGE analysis	$\checkmark$	$\times$	$\checkmark$	$\checkmark$
Dynamic number of analyses	$\checkmark$	$\times$	$\times$	$\checkmark$
Individual significance thresholds	$\times$	$\times$	$\times$	$\checkmark$
DGE analysis result	$\checkmark$	$\checkmark$	$\checkmark$	$\checkmark$
Compare DGE analyses	$\times$	$\times$	$\times$	$\checkmark$
GO based enrichment analyses	$\times$	$\times$	$\times$	$\checkmark$
Compare GO anlayses	$\times$	$\times$	$\times$	$\checkmark$
Web application	$\times$	$\checkmark$	$\checkmark$	$\checkmark$
Reproducibility	$\times$	$\checkmark$	$\checkmark$	$\checkmark$

### Benefits and extensions

Our web application provides a platform for all steps of gene expression analysis from different microarray technologies. Additionally, GO enrichment analyses and comparison of analyses are enabled. All steps are implemented in a user-friendly way and visualize the results using different plots. Furthermore, by providing a web application, our tool does not require any installations and maintenance from the users and works platform independent.

Our framework enables novel analyses that scientists without in-depth programming skills can perform themselves. For example, data produced with different technologies can be easily compared — hence, avoiding the need to rerun experiments with different tools. In addition, gene expression data can also be compared between organisms, as demonstrated in chapter:case-study-ii, allowing early conclusions about the validity of animal experiments. Because of these advantages, we initially focused on the older microarray technology. Based on various studies, the results are of similar quality as from the newer next generation sequencing (NGS) methods. Moreover, microarray technologies are still necessary to use for some applications [8, 50–52]. Therefore, these data should be usable further and comparable with new data generated by novel technologies, such as NGS (e.g. RNA-seq).

Comparing microarray and NGS data can be helpful for methodological reasons, such as evaluating the accuracy and reproducibility of the different technologies. By comparing the results obtained with both methods, researchers can identify any inconsistencies or errors and work to improve their experimental protocols. However, only data generated with Affymetrix and Agilent microarrays can be analyzed with our application. Nevertheless, the importance of NGS as an essential tool to study genetic variation, gene expression and epigenetic changes should not be underestimated. Therefore, we plan to extend our application to enable the analysis of these data as well. The modular design of the program code allows for easy extension to this end or to add further enrichment analyses.

## Conclusion

Scientists are often challenged to analyse gene expression data from their experiments and compare the results with other studies. Among other things, the comparison of gene expression between different organisms is of particular interest, for example due to the transferability of animal experiments to humans. We have presented a web application that allows the user to analyse microarray gene expression data autonomously, without the need for programming skills. While our application offers a wide range of analysis options, for example through different preprocessing methods, it also offers default values or more information for the user as far as possible. The workflow of the web application is divided into five main steps, to which the UI is adapted. By comparing DGE and GO analyses, different preprocessing approaches can be compared. Its main feature is that it allows for a cross-platform and cross-species comparison of various studies, even from different organisms or technologies.

In contrast to existing software, our application offers several advantages. First, it is web-based, so there are no system requirements or installation difficulties. It allows the analysis of Affymetrix as well as one-channel and two-channel Agilent data, with the analysis steps being almost identical in each case – hence offering flexible analysis options. The possibility of GO enrichment analyses and the comparison of DGE and GO analyses is also an extension to most existing applications.

Currently, we are developing an extension for the analysis of RNA-seq data in the application as well. Thus, our web application would provide an all-in-one platform for gene expression analysis. In addition, enabling further enrichment analyses, for example pathway analyses, would be of interest for future work. In future work we also aim to look into applications of machine learning on genomic data as for example surveyed in [53].

## Availability and requirements

**Table:** 

Project name	Genealyzer
Project home page	https://genealyzer.item.fraunhofer.de/
Operating system(s)	Platform independent
Programming language	R v4.1.2
Other requirements	Up-to-date web browser
	Test credentials are
	username: demo
	password: Gen?eITEMaly-zer
License	limited non-commercial research license
	commercial extended research use: requires license
Any restrictions to use by non-academics	licence needed

## Data Availability

The data used in case study I is available on ArrayExpress with accession code E-MTAB-2967. The data used in case study II is available on the GEO platform with the series numbers GSM3614926 and GSM4093523.

## Abbreviations

CD: Crohn’s disease
CEL: File extension of files generated with Affymetrix microarrays
DEG: Differentially expressed genes
DGE: Differential gene expression
GEO: Gene expression omnibus
GO: Gene ontology
GSE: GEO series accession number
NGS: Next generation sequencing
PCA: Principal component analysis
PCLS: Precision cut lung slices
SDRF: Sample and data relationship format
TAC: Transcriptome analysis console
UC: Ulcerative colitis
UI: User interface

## Footnotes

1 https://gepat.sourceforge.net/installation.htm
